# No measurable adverse effects of Lassa, Morogoro and Gairo arenaviruses on their rodent reservoir host in natural conditions

**DOI:** 10.1186/s13071-017-2146-0

**Published:** 2017-04-27

**Authors:** Joachim Mariën, Benny Borremans, Sophie Gryseels, Barré Soropogui, Luc De Bruyn, Gédéon Ngiala Bongo, Beate Becker-Ziaja, Joëlle Goüy de Bellocq, Stephan Günther, N’Faly Magassouba, Herwig Leirs, Elisabeth Fichet-Calvet

**Affiliations:** 1 Evolutionary Ecology Group, University of Antwerp, Antwerp, Belgium; 2 Projet des Fièvre Hémorragiques en Guinée, Hôpital Donka, Conakry, Guinea; 3 Bernhard-Nocht-Institute for Tropical Medicine, Hamburg, Germany; 4 Institute of Vertebrate Biology, Research Facility Studenec, The Czech Academy of Sciences, Brno, Czech Republic; 5 Department of Ecology and Evolutionary Biology, University of California, Los Angeles, USA; 6 Department of Ecology and Evolutionary Biology, University of Arizona, Tucson, USA; 7 Department of Biology, University of Kinshasa, P.O. Box. 190, Kinshasa XI, Democratic Republic of the Congo

**Keywords:** Arenavirus, Lassa virus, Morogoro virus, Gairo virus, *Mastomys natalensis*, Rodent-borne disease, Host-pathogen interaction, Reservoir host

## Abstract

**Background:**

In order to optimize net transmission success, parasites are hypothesized to evolve towards causing minimal damage to their reservoir host while obtaining high shedding rates. For many parasite species however this paradigm has not been tested, and conflicting results have been found regarding the effect of arenaviruses on their rodent host species. The rodent *Mastomys natalensis* is the natural reservoir host of several arenaviruses, including Lassa virus that is known to cause Lassa haemorrhagic fever in humans. Here, we examined the effect of three arenaviruses (Gairo, Morogoro and Lassa virus) on four parameters of wild-caught *Mastomys natalensis*: body mass, head-body length, sexual maturity and fertility. After correcting for the effect of age, we compared these parameters between arenavirus-positive (arenavirus RNA or antibody) and negative animals using data from different field studies in Guinea (Lassa virus) and Tanzania (Morogoro and Gairo viruses).

**Results:**

Although the sample sizes of our studies (1297, 749 and 259 animals respectively) were large enough to statistically detect small differences in body conditions, we did not observe any adverse effects of these viruses on *Mastomys natalensis*. We did find that sexual maturity was significantly positively related with Lassa virus antibody presence until a certain age, and with Gairo virus antibody presence in general. Gairo virus antibody-positive animals were also significantly heavier and larger than antibody-free animals.

**Conclusion:**

Together, these results suggest that the pathogenicity of arenaviruses is not severe in *M. natalensis*, which is likely to be an adaptation of these viruses to optimize transmission success. They also suggest that sexual behaviour might increase the probability of *M. natalensis* to become infected with arenaviruses.

**Electronic supplementary material:**

The online version of this article (doi:10.1186/s13071-017-2146-0) contains supplementary material, which is available to authorized users.

## Background

The Natal multimammate mouse, *Mastomys natalensis* (Smith, 1834), is the natural host of six known Old World arenaviruses (Arenaviridae, *Mammarenavirus*): Lassa (LASV) and a Mobala-like virus in West Africa, and Morogoro (MORV), Mopeia, Luna and Gairo viruses (GAIV) in Southern East Africa [1–7]. Of these six viruses, only LASV is known to cause Lassa haemorrhagic fever in humans, who can become infected by close contact with infectious rodents or their excretions [1]. Annually around 200,000 people are affected, with a fatality rate of 1–2% [8–10].

While the pathogenic effects of LASV on humans are well-documented [11, 12], little attention has been paid to the effects on its reservoir host. Nevertheless, knowledge of how parasites affect their reservoir hosts is relevant in the study of wildlife diseases, as this helps to understand how host population dynamics or behaviour affect the spread of the parasite through the population [13]. In general, in order to optimize net transmission success, parasites face a trade-off between within-host reproduction, which requires the utilisation of host resources, and between-host transmission probability, which requires the hosts to be sufficiently healthy and long-lived to encounter new susceptible individuals [14–16]. Usually, a long evolutionary history in a particular host results in low virulence towards the natural reservoir [17, 18].

Most information about arenavirus pathogenicity is derived from inoculation studies on laboratory house mice (*Mus musculus*) with lymphocytic choriomeningitis virus (LCMV), an Old World arenavirus that shares 60% amino acid similarity with LASV [19]. House mice are the reservoir hosts of LCMV and most LCMV strains result in symptom-free infection, despite high levels of viral growth [20, 21]. Nevertheless, severe disease (including meningitis, reduced growth and death) did develop for some mouse breed - LCMV strain combinations [22–25]. Variation in pathogenicity was also noted in vesper mice (*Calomys callosus* and *Calomys musculinus*) which are the natural hosts of respectively New World Machupo and Junin arenaviruses. Inoculation of *C. callosus* with Machupo virus resulted in a dual response in which about half of the infected animals became immunocompetent with transient viremia and no disease symptoms, whereas the other half became immunotolerant and exhibited persistent viremia and reduced body size, fertility and lifespan [26]. Inoculation with Junin virus caused a similar response in *C. musculinus*, where adults remained asymptomatic while juveniles experienced reduced body size, fertility and lifespan [27, 28].

Few studies have examined the effects of arenaviruses in *M. natalensis*. Two inoculation studies, on LASV and MORV, showed no or only mild overt disease symptoms in their rodent host [29, 30]. MORV inoculations in adult wild type *M. natalensis* caused significant temporary 7% weight loss 10 days post-infection in about half of the animals. Although these animals recovered completely in the laboratory, such weight loss in natural circumstances might have significant consequences for survival probability. In contrast to MORV inoculation studies, two field studies found that LASV reduces growth and fecundity of *M. natalensis*, and suggested that LASV severely affects its rodent host [31, 32]. These studies should however be interpreted with caution, as the first provided limited statistical proof, while the latter used body mass category as an estimate to correct for an age effect on prevalence, which is a potentially unreliable proxy [33].

Knowledge of the exact age is essential to estimate pathogenicity effects, since age correlates with many morphometric and sexual parameters. More importantly, in case of acute infections with long-term presence of antibodies (observed for LASV-related arenaviruses in *M. natalensis* [2, 34, 35]), older animals are more likely to have already encountered the infection, and developed antibodies, during their lifetime simply due to the longer time window they have been alive. In this situation, the probability of detecting active, recent infection decreases with age, as more susceptible (antibody-negative) animals are available in the younger age categories compared to the older categories [35]. Therefore, if one wants to investigate the effect of infection on an age-dependent host variable (e.g. body mass, head-body length or sexual maturity), the effect of age should be disentangled from that of the variable [36].

In this paper, we want to systematically analyse data from three different *M. natalensis*-borne arenaviruses in the same way, in order to confidently test whether infection affects the host in natural conditions. We will test the relationships between arenavirus (LASV, MORV and GAIV) infection and body mass, body length, sexual maturity and fertility. We correct for the effects of age by using eye lens weight (ELW), which is known to be a good proxy for age because the eye lens is continuously growing, independent of environmental factors [37–41].

We previously investigated the relationships between LASV infection and several parameters such as village, habitat, season, age and abundance in Guinea [42–44]. Using more recent data, we here expand this analysis to body condition and increase the dataset from 553 to 1,297 individuals. For MORV, a previous investigation on its effect on *M. natalensis* body condition was performed on a small dataset (*n* = 171) during one season and locality only, where no significant relationship between MORV infection and body mass index was found, although a model including body mass index did result in the best prediction of active MORV infection [35]. Here we supplement this dataset with new data in order to arrive at a sample size of 749 individuals. Our recent work on GAIV tested the relationship between infection and host condition (using infection status as dependent variable) [2], but here we re-analyse these data in exactly the same way as the other two viruses (using body condition as dependent variable), as this allows a direct comparison between the three viruses.

## Methods

### Guinean study sites, rodent sampling, and LASV screening of rodents

For the examination of the relationships between LASV and *M. natalensis* body condition, we re-analysed data from previous publications [42, 44, 45] and also present new data. Here, we provide a summary of the data collection procedures, but refer to the mentioned publications for more details. Rodents were captured in thirteen villages with reported human LASV cases in Upper Guinea during an intermittent time span between 2003 and 2015. Traps were placed both indoors and outdoors during the rainy and dry season. Captured rodents were humanely killed and measured morphometrically. Sexual maturity of females was determined by pregnancy, lactation status and by signs of scars in a large uterus (width > 4 mm). Body mass of pregnant females was adjusted for foetus + uterus mass. Fertility of pregnant females was determined by counting the number of foetuses inside the uterus. Males were considered sexually mature if the vesiculae seminales were swollen and their surface exceeded 100 mm^2^. Eyes were preserved in 10% formalin, the lenses were extracted, cleaned and dried for 2 h at 100 °C, and then weighed to the nearest 0.1 mg. Whole blood, spleen, kidney, and liver were collected during the autopsies and stored in liquid nitrogen or in -20 °C. Detection of LASV viral RNA (vRNA) was performed and examined by RT-PCR on blood and spleen of 1,298 *M. natalensis* [46, 47]. Detection of anti-LASV antibodies was performed on 1,139 *M. natalensis* and examined by indirect immunofluorescence assay [48, 49]. For this assay, Vero cells infected with LASV strain Bantou were spread on immunofluorescence slides, air dried, and acetone-fixed. Antibodies in positive samples would then bind to antigens presented by the Vero cells and be visualized with anti-mouse IgG-FITC secondary antibodies. In total, 168 (13%) LASV vRNA and 318 (28%) antibody positive individuals were detected.

### Tanzanian study sites, rodent sampling, and MORV or GAIV screening of rodents

For the examination of the relationships between MORV or GAIV infection and *M. natalensis* body condition, we (re-)analysed data from previous publications [2, 3, 35] and here present new antibody data from the nine localities where MORV circulates as described in [3]. Rodents were trapped outdoors in the Morogoro region in Tanzania intermittently between 2008 and 2012. Captured rodents were humanely killed and identified morphometrically. Reproductive status was measured by investigating external characteristics of the reproductive organs, as described in [39]. Eye lens weight (ELW) was measured using the method described for the LASV studies, except that they were dried for > 2 h at 80 °C. Organs were preserved in RNAlater for viral detection, and blood on serobuvard filter paper (±15 μl/punch; Serobuvard, LDA 22, Zoopole, France) for antibody detection. In total, 743 and 259 *M. natalensis* were tested for respectively MORV and GAIV vRNA using RT-PCR reaction [5, 46], and 683 and 259 *M. natalensis* were tested for the presence of anti-MORV and anti-GAIV antibodies using indirect immunofluorescence assay. Vero cells infected with MORV strain 3017/2004 were spread on immunofluorescence slides, air dried, acetone-fixed, and visualised as described for LASV. For MORV and GAIV respectively, 48 (6%) and 29 (11%) animals tested vRNA positive, and 73 (11%) and 25 (9%) animals tested antibody positive [2, 3, 35].

### Statistical analysis

We tested the relationships between arenavirus infection and four different measures of *M. natalensis*’ body condition: body mass, head-body (HB) length and sexual maturity for all viruses, and fertility for LASV only with linear mixed models and generalised linear mixed models. For all the investigated parameters it was important to disentangle the effect of age from that of the measured variable. For this reason, we developed models with one of the parameters of interest as dependent variable and ELW (proxy for age), infection status (RNA/antibody positive or negative) and their interaction as independent fixed variables. We also included sex, season (rainy/dry) and their interaction with ELW and infection status as independent fixed variables, and year and village as independent random variables to correct for effects of year and location. We log-transformed body mass, HB length and ELW since we observed a linear relation between log(body mass) and log(HB length) on the one hand and log(ELW) on the other hand. The relationships to sexual maturity (i.e. the probability of being sexually active, 1 = active, 0 = inactive) were assessed assuming a binomial distribution (logit-link function). The relationships to fertility (i.e. number of fetuses inside the uterus, only for sexually mature females) were assessed assuming a Poisson distribution (log-link function). We performed separate analyses for three different infection statuses: (i) only arenavirus RNA, (ii) only antibody, and (iii) either arenavirus RNA or antibody presence, or both.

In one of the 10 villages investigated in 2013 in Upper Guinea, no LASV was detected. We therefore compared the effect of infection in *M. natalensis* living in this LASV negative village (Tambaya, *n* = 50) to those living in the nearest LASV positive village (Brissa, *n* = 62). Season and year were removed from this model as these animals were collected in the same period, and LASV village (positive/negative) was implemented as an independent fixed variable.

We used the lmer and glmer functions of the *lme4* package (version 1.1-7) of the R statistical software version 3.3.0 [50] to run the mixed models. When fitting the models, we started with the fully parameterised models (all interactions between the independent fixed variables) and sequentially dropped variables that had the highest insignificant p-values. Significance was tested with the likelihood ratio test (assuming a *χ* ^2^ residual distribution).

## Results

Because the effects of the parameters ELW, infection status and their interactions on the body condition variables (body mass, head-body length, sexual maturity and fertility) were of main interest in this study, we summarized these in Table 1. The effects of other parameters (sex, season and their interaction) on the body condition variables, were presented in Additional file 1: Tables S1 and S2).

**Table 1:** Effects of age (Eye lens weight - ELW) and viral infection (viral RNA - vRNA and antibody - AB) on body mass, head-body length, sexual maturity and fertility in *M. natalensis* infected by Lassa (LASV), Morogoro (MORV) and Gairo (GAIV) viruses

			ELW		vRNA		Interaction vRNA × ELW		AB		Interaction		vRNA or AB		Interaction	
											AB × ELW				vRNA or AB × ELW	
Virus	Predicted	*n*	${\upchi}_1^2$	*P*	${\upchi}_1^2$	*P*	${\upchi}_1^2$	*P*	${\upchi}_1^2$	*P*	${\upchi}_1^2$	*P*	${\upchi}_1^2$	*P*	${\upchi}_1^2$	*P*
LASV	Body mass	1,297	1670.30	**< 0.01**	0.53	0.46	0.11	0.74	1.78	0.18	0.49	0.49	2.08	0.14	0.13	0.71
	Head-body length	1,296	1751.80	**< 0.01**	0.16	0.68	0.09	0.75	0.05	0.81	0.15	0.70	0.29	0.65	<0.01	0.97
	Sexual maturity	1,297	345.21	**< 0.01**	1.72	0.54	0.32	0.57	np	np	5.42	**0.02**	0.01	0.91	2.78	0.10
	Fertility	151	1.84	0.17	2.14	0.14	0.21	0.65	2.14	0.14	0.03	0.85	0.10	0.75	0.08	0.78
MORV	Body mass	747	331.22	**< 0.01**	0.01	0.94	0.85	0.36	0.02	0.90	0.85	0.35	< 0.01	0.95	1.26	0.26
	Head-body length	514	127.46	**< 0.01**	0.04	0.83	1.50	0.22	0.01	0.94	0.03	0.87	< 0.01	0.97	0.13	0.72
	Sexual maturity	749	37.99	**< 0.01**	0.16	0.69	0.27	0.60	0.08	0.77	0.01	0.93	< 0.01	0.96	0.03	0.84
GAIV	Body mass	258	176.09	**< 0.01**	0.03	0.86	0.52	0.46	13.28	**< 0.01**	0.01	0.93	8.60	**< 0.01**	< 0.01	0.99
	Head-body length	254	175.00	**< 0.01**	0.14	0.71	0.29	0.59	8.45	**< 0.01**	0.95	0.33	6.23	**0.01**	0.23	0.63
	Sexual maturity	256	25.90	**< 0.01**	0.09	0.76	0.12	0.72	8.57	**< 0.01**	0.49	0.48	6.14	**0.01**	0.53	0.46

*Abbreviations: n* number of sampled *M. natalensis*; *np* not possible to calculate because of significant interaction; ${\upchi}_1^2$, chi-squared value; *P, P-value*

The last two columns represent analyses of grouped animals that were either vRNA or antibody positive. Bold *P-values* are statistically significant

**Fig. 1:**
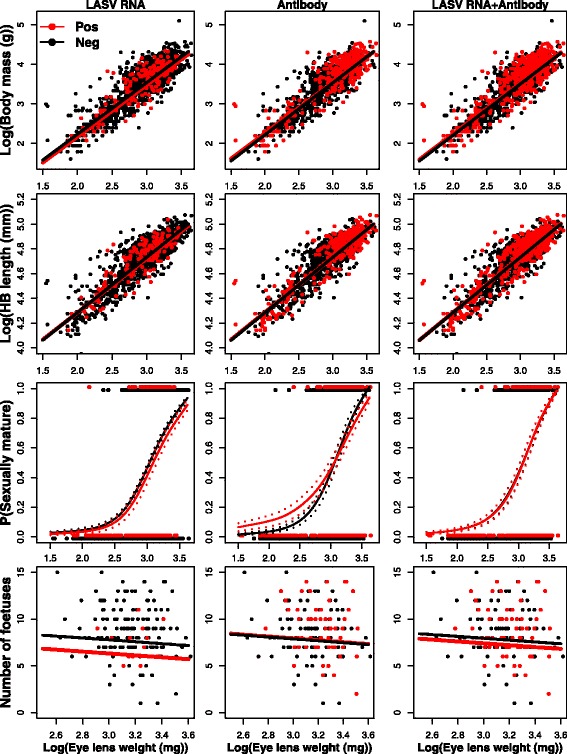
Relationships between body mass, head-body length, sexual maturity (1 = active and 0 = inactive), fertility (# foetuses) and eye lens weight (as a proxy for age) in *M. natalensis* infected by Lassa virus*. Red* = positive, *black* = negative*.* Dashed lines represent standard errors on the predicted probabilities

**Fig. 2:**
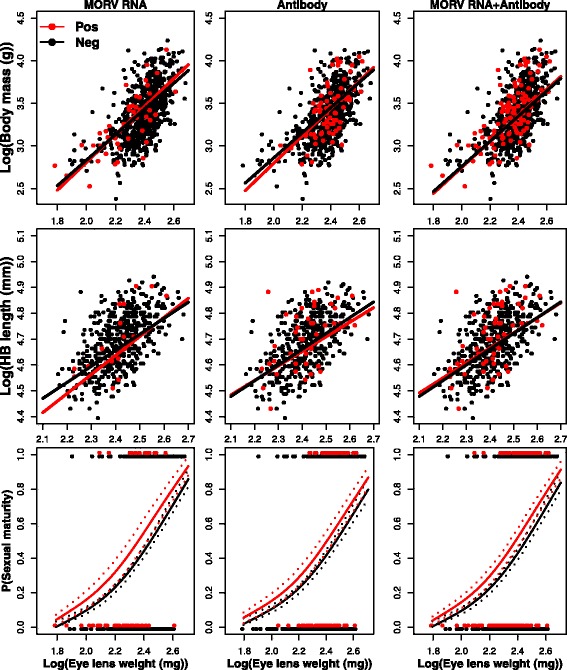
Relationships between body mass, head-body length, sexual maturity (1 = active and 0 = inactive) and eye lens weight (as a proxy for age) in *M. natalensis* infected by Morogoro virus*. Red* = positive, *black* = negative*.* Dashed lines represent standard errors on the predicted probabilities

**Fig. 3:**
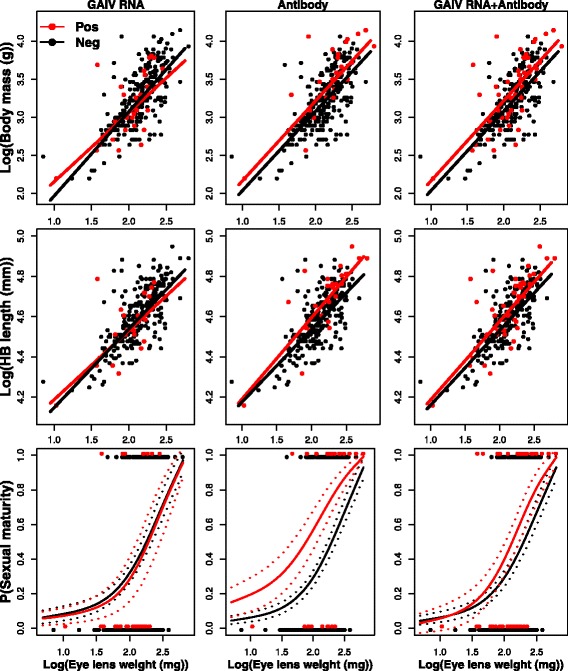
Relationships between body mass, head-body length, sexual maturity (1 = active and 0 = inactive) and eye lens weight (as a proxy for age) in *M. natalensis* infected by Gairo virus*. Red* = positive, *black* = negative*.* Dashed lines represent standard errors on the predicted probabilities

The three body condition predictor variables (body mass, head-body length and sexual maturity) were all highly correlated with age in the three investigated populations of *M. natalensis* (${\chi}_1^2$ > 25.90, *P* < 0.01) (Figs. 1, 2 and 3). As reported previously, presence of either vRNA or antibody is also correlated significantly with age for all three viruses (${\chi}_1^2$ > 4.75, *P* < 0.03) (Additional file 1: Table S3).

Correcting for this general age effect, we observed almost no effect of either active or past arenavirus infection on body condition (Table 1). For none of the investigated viruses, presence of vRNA was significantly related to any of the predictor variables, nor were any of the interactions between vRNA presence and ELW significantly related to any of the predictor variables (Table 1).

Antibody presence was not significantly related to any of the predictor variables for MORV. For GAIV, we found significant relationships between antibody presence and all three body condition predictor variables (Table 1, Fig. 3): antibody-positive *M. natalensis* were significantly heavier (${\chi}_1^2$ = 13.28, *P* < 0.01) and larger (${\chi}_1^2$ = 8.45, *P* < 0.01) than antibody negative ones, and had a significantly higher probability of being sexually mature (${\chi}_1^2$ = 8.57, *P* < 0.01). For LASV, antibody presence alone was not significantly related to body mass and HB length. The relationship between LASV antibodies and sexual maturity could not be assessed due to a significant interaction between antibody and ELW (${\chi}_1^2$ = 5.42, *P* = 0.02), in which young antibody-positive *M. natalensis* had a higher probability of being sexually mature than antibody-negative animals (Fig. 1). This difference disappeared for older animals where more animals are sexually mature (${\chi}_1^2$ = 1.86, *P* = 0.06). Interactions between antibody presence and ELW had no significant effect on any of the body condition variables in the case of MORV and GAIV.

The presence of either vRNA, antibody or both (indicative of successful infection at any point in the past) was not significantly related to any of the predictor variables for MORV and LASV, nor was the interaction between this definition of infection status and ELW significantly related to any of the predictor variables. For GAIV, there were significant relationships between the infection status and all three body condition predictor variables: past or recent infected animals where significantly heavier (${\chi}_1^2$ = 8.60, *P* < 0.01) and larger (${\chi}_1^2$ = 6.23, *P* = 0.01), and had a higher probability of being sexually mature (${\chi}_1^2$ = 6.14, *P* = 0.01) than animals that were never infected (Table 1, Fig. 3).

We could analyse the relationship between infection and fertility for the LASV dataset only. Fertility in females as measured by the number of foetuses was independent of age (${\chi}_1^2$ = 1.84, *P* = 0.17), and no significant difference in number of foetuses was observed between LASV RNA/antibody-positive and negative pregnant females (${\chi}_1^2$ < 2.14, *P* > 0.14) (Table 1). For LASV, we could also compare a LASV-negative village (Tambaya) with its nearest LASV-positive village (Brissa). We did not find any significant differences in body mass, head-body length or sexual maturity between *M. natalensis* in the two villages (${\chi}_1^2$ < 2.62, *P* > 0.09) (Additional file 1: Table S4; Additional file 2: Figure S1).

## Discussion

We did not observe any significant negative associations between arenavirus infection and *M. natalensis* morphometric or sexual parameters, suggesting that these viruses are harmless for the reservoir hosts in natural conditions. Our results are in contrast with the studies of Demartini et al. [31] and Lalis et al*.* [32]. Demartini et al. [31] found that LASV infections resulted in less heavy animals and inflammatory lesions. As these authors noted, important limitations of their study were the small sample size (*n* = 28) and lack of information about the animals’ age. While Lalis et al. [32] also found no association between LASV presence and host body mass, they did find that LASV positive animals had a significantly smaller body size, skull and size of the reproductive organs in the adult category. Their adult category based on the body mass (> 40 g) was highly variable and can cover a range of 40–97 g (ELW = 15–38 mg in figure 5 in EFC et al. [43]). Among this body weight class, 73% (47/64) of the LASV positive animals were belonging to the youngest ELW class (15–25 mg). We can suggest that their 30 LASV positive adult samples were possibly younger, smaller and more inactive sexually than their 38 LASV negative ones.

Our results are consistent with the laboratory inoculation studies of Walker et al. [30] and Borremans et al. [29], who observed no or only mild disease symptoms of arenaviruses in *M. natalensis*. Walker et al. [30] showed that neonatal animals inoculated with LASV exhibited a persistent tolerant infection with no histopathological signs of disease. Nevertheless, some animals infected as adults developed moderate (not deadly) meningoencephalitis. Borremans et al. [29] found that inoculation of *M. natalensis* with MORV could decrease the weight of animals between days 7 and 15 post-infection (±7% of normal weight in 40% of animals), but this decrease was only temporary and did not further affect the animals’ growth rates.

It should be noted that we did not provide proof that these viruses never affect their reservoir host. For example, our data do not exclude that some animals become sick, die quickly from disease and thus are not trapped during field studies. Also, we only checked four host parameters, while viruses might affect their host in many different ways (e.g. differences in hormone levels, brain size or temperature may be found [21]). Such complex relationships between parasite infection and host fitness were indeed observed in other rodent-borne diseases. For example, infection of Puumala hantavirus in bank voles (*Myodes glareolus*) was initially assumed to be asymptomatic [51–53] but recent long-term field studies observed negative effects on the survival probability and an equivocal impact on the fecundity of females [36, 54, 55]. An important difference between Old World arenaviruses and hantaviruses, however, is that the latter causes persistent infections in their reservoir hosts [56], while the former infects their hosts mainly acutely [29, 43]. The lower survival probability caused by hantaviruses could therefore perhaps be explained by an accumulation of small deleterious effects over time, which might not be the case during the relatively short arenavirus infections. Nevertheless, performing survival analyses would be a logical next step to further explore possible effects of arenaviruses in *M. natalensis*, and could be done by investigating long-term capture-mark-recapture data.

In general, our study supports the hypothesis that the pathogenicity of arenaviruses is low in their natural hosts. This is likely an adaptation of these viruses to increase the probability of transmitting the infection to other individuals. In contrast, highly pathogenic viruses might fail in transmission because they reduce the host’s infectious period, or reduce its contact rate as a trade-off for energy to eradicate the virus [13, 57]. A non-pathogenic lifestyle would be especially beneficial for MORV, since MORV is endemic in populations of *M. natalensis* that fluctuate heavily between seasons, generally ranging from 20 to 300 individuals per hectare [5, 58]. Mathematical models suggest that these fluctuations impose a severe impediment for MORV persistence, because during the low density periods, the transmission chain is heavily dependent on a few susceptible and infectious individuals only [59]. Nevertheless, MORV can persist in these highly fluctuating populations, and can be detected even at very low densities [35]. In order to survive these bottlenecks, we assume that adaptation has driven MORV to a low-pathogenic lifestyle. A similar situation is suggested for the persistence of Junin arenavirus in populations of *Calomys musculinus*. Vitullo & Merani [27, 60] found that Junin virus did not affect the fitness of adult *C. musculinus* and, based on a mathematical model, they assumed that these unaffected animals are a prerequisite for viral persistence.

After correcting for the effect of age, we found that GAIV antibody-positive *M. natalensis* were significantly heavier, larger and reached sexual maturity earlier than antibody-free rodents. We also found that LASV antibody-positive rodents reached sexual maturity earlier until a certain age. These results are compatible with the hypothesis that sexual behaviour increases the probability of becoming infected with arenaviruses, and that sexual transmission could therefore be an important mechanism for the spread of arenaviruses in populations of *M. natalensis*. Although this relationship was not found for LASV in older animals or for MORV at all, sexual transmission would indeed be a highly beneficial transmission route for arenaviruses. Arenaviruses are assumed to be transmitted horizontally (at least partly) by as yet unconfirmed pathways [2, 35, 42, 44], and *M. natalensis* has a promiscuous mating system in which both males and females endeavour to copulate with numerous mates [39, 61, 62]. Sexual transmission is also assumed to be important in other virus-rodent host systems. Cage experiments showed that the transmission rate of Machupo arenavirus in *C. callosus* was considerably higher in opposite sex pairs than in same sex pairs, and high concentrations of virus and antibody were found in the reproductive organs [26]. In natural populations of bank voles infected with Puumala hantavirus, seroconversion was observed to occur more frequently during the reproductive season and in sexually active individuals [51]. However, a recent long-term field study showed that seroconversion rates of bank voles are significantly higher outside the reproductive season, which occurs in winter [63]. This last result supports the hypothesis of an active indirect transmission through contaminated soil during wet and cold conditions [64]. The two conflicting results highlight that patterns within the same parasite-host system can differ considerably due to different environmental conditions.

Otherwise, the observed relation between antibodies and sexual maturity in our study might also be explained by the hypothesis that arenavirus replication enhances sexual maturity or behaviour of its host. This might be likely, as it has been shown that LCMV can severely disrupt hormonal regulation in laboratory mice [25, 65, 66]. Similarly, Kallio et al. [36] suggest that infection with Puumala hantavirus enhances breeding in young female bank voles, but not in old ones.

## Conclusion

We do not find any evidence that *M. natalensis*-borne arenaviruses negatively affect their natural host in natural circumstances. While adverse effects that lead to low capture probability in a subset of the infected population cannot be ruled out, our results suggest that the pathogenicity of arenaviruses is low in *M. natalensis* in general. This implies that these viruses do not significantly affect the population dynamics of *M. natalensis*. Furthermore, as our study indicates that sexual behaviour can be important for arenavirus transmission in natural populations of *M. natalensis*, it would be interesting to determine the extent to which sexual transmission occurs. If an important mode of transmission, and considering mating frequency could be expected to be density-dependent in *M. natalensis*, control measures aimed at reducing the abundance of rodents during the breeding season may be highly effective for virus eradication.

## Additional files

Additional file 1: Table S1. Effects of sex and viral infection (viral RNA - vRNA and antibody - AB) on body mass, head-body length, sexual maturity and fertility in *M. natalensis* infected by Lassa (LASV), Morogoro (MORV) and Gairo (GAIV) viruses. Estimate effects were shown on a log-scale to allow linear comparison. The last two columns represent animals that were vRNA or antibody positive. (*n* = number of sampled *M. natalensis*; *statistically significant, NP, not possible to calculate because of significant interaction with ELW). **Table S2.** Effects of season and viral infection (viral RNA - vRNA and antibody - AB) on body mass, head-body length, sexual maturity and fertility in *M. natalensis* infected by Lassa (LASV), Morogoro (MORV) and Gairo (GAIV) viruses. Estimate effects were shown on a log-scale to allow linear comparison. The last two columns represent animals that were vRNA or antibody positive. (*n* = number of sampled *M. natalensis*; *statistically significant, NP^a^, not possible to calculate because of significant interaction with ELW, NP^b^, not possible to calculate because there were not enough data sampled in one of the two seasons). **Table S3.** Relation between viral infection (viral RNA - vRNA and antibody - AB) and age (eye lens weight) in *M. natalensis* infected by Lassa (LASV), Morogoro (MORV) and Gairo (GAIV) viruses. **Table S4.** Effects on body mass, head-body length and sexual maturity of *M. natalensis* between a Lassa virus (LASV) positive and negative village (Brissa versus Tambaya) (*n* = number of sampled *M. natalensis*). (DOCX 27 kb)

Additional file 2: Figure S1. Relationships between body mass, head-body length, sexual maturity (1 = active and 0 = inactive) and Eye lens weight (as a proxy for age) in *M. natalensis* infected by Lassa virus (LASV). Red = LASV positive village (Brissa), black = LASV negative village (Tambaya). Dashed lines represent standard errors on the predicted probabilities. (PDF 100 kb)

## Abbreviations

ELW: Eye lens weight
GAIV: Gairo virus
HB: Head body
LASV: Lassa virus
LCMV: Lymphocytic choriomeningitis virus
MORV: Morogoro virus
vRNA: viral RNA
