# Supplementary material for: No measurable adverse effects of Lassa, Morogoro and Gairo arenaviruses on their rodent reservoir host in natural conditions
Source: Parasit Vectors. 2017 Apr 27;10:210. doi: 10.1186/s13071-017-2146-0 (PMC5408478; doi:10.1186/s13071-017-2146-0)
Supplement: Supplementary file 1 — Effects of sex and viral infection (viral RNA - vRNA and antibody - AB) on body mass, head-body length, sexual maturity and fertility in M. natalensis infected by Lassa (LASV), Morogoro (MORV) and Gairo (GAIV) viruses. Estimate effects were shown on a log-scale to allow linear comparison. The last two columns represent animals that were vRNA or antibody positive. (n = number of sampled M. natalensis; *statistically significant, NP, not possible to calculate because of significant interaction with ELW). Table S2. Effects of season and viral infection (viral RNA - vRNA and antibody - AB) on body mass, head-body length, sexual maturity and fertility in M. natalensis infected by Lassa (LASV), Morogoro (MORV) and Gairo (GAIV) viruses. Estimate effects were shown on a log-scale to allow linear comparison. The last two columns represent animals that were vRNA or antibody positive. (n = number of sampled M. natalensis; *statistically significant, NPa, not possible to calculate because of significant interaction with ELW, NPb, not possible to calculate because there were not enough data sampled in one of the two seasons). Table S3. Relation between viral infection (viral RNA - vRNA and antibody - AB) and age (eye lens weight) in M. natalensis infected by Lassa (LASV), Morogoro (MORV) and Gairo (GAIV) viruses. Table S4. Effects on body mass, head-body length and sexual maturity of M. natalensis between a Lassa virus (LASV) positive and negative village (Brissa versus Tambaya) (n = number of sampled M. natalensis). (DOCX 27 kb) [file 13071_2017_2146_MOESM1_ESM.docx]

**Additional file 1**

**Additional file 1: Table S1**

Effects of sex and viral infection (viral RNA - vRNA and antibody - AB) on body mass, head-body length, sexual maturity and fertility in *M. natalensis* infected by Lassa (LASV), Morogoro (MORV) and Gairo (GAIV) viruses. The last two columns represent animals that were vRNA or antibody positive. *Abbreviations*: n, number of sampled *M. natalensis*; *, statistically significant; NP^a^, not possible to calculate because of significant interaction with ELW; NP^b^, not possible to calculate because there were not enough data sampled in one of the two seasons.

|  | |  | **sex** | | **Interaction**  **sex x ELW** | | **Interaction**  **Sex x vRNA** | | **Interaction sex x AB** | | **Interaction**  **sex x vRNA or AB** | |
| --- | --- | --- | --- | --- | --- | --- | --- | --- | --- | --- | --- | --- |
| Virus | *Predicted* | *n* | $\chi_{1}^{2}$ | *p-value* | $\chi_{1}^{2}$ | *p-value* | $\chi_{1}^{2}$ | *p-value* | $\chi_{1}^{2}$ | *p-value* | $\chi_{1}^{2}$ | *p-value* |
| **LASV** | Body mass | 1297 | NP | NP | 12.37 | <0.01* | 1.46 | 0.23 | 0.33 | 0.56 | 2.01 | 0.15 |
|  | Head-body length | 1296 | NP | NP | 7.96 | <0.01* | 2.56 | 0.11 | 0.05 | 0.87 | 1.07 | 0.30 |
|  | Sexual maturity | 1297 | NP | NP | 30.90 | <0.01* | 1.62 | 0.20 | 0.14 | 0.71 | 0.34 | 0.56 |
|  | Body mass | 747 | 75.50 | <0.01* | 2.02 | 0.15 | 1.42 | 0.23 | 0.33 | 0.56 | 0.59 | 0.44 |
| **MORV** | Head-body length | 514 | NP | NP | 12.89 | <0.01* | 1.78 | 0.18 | 0.68 | 0.41 | 1.10 | 0.29 |
|  | Sexual maturity | 749 | 125.06 | <0.01* | 0.68 | 0.41 | 0.93 | 0.33 | 0.74 | 0.38 | 0.03 | 0.87 |
|  | Body mass | 258 | 0.84 | 0.36 | 2.97 | 0.09 | 0.02 | 0.90 | 0.88 | 0.34 | 0.35 | 0.56 |
| **GAIV** | Head-body length | 254 | 0.59 | 0.44 | 2.22 | 0.13 | 0.42 | 0.51 | 0.43 | 0.51 | 0.96 | 0.33 |
|  | Sexual maturity | 259 | 12.15 | <0.01* | 1.67 | 0.19 | 0.05 | 0.94 | 0.02 | 0.87 | 0.03 | 0.86 |

**Additional file 1: Table S2**

Effects of season and viral infection (viral RNA - vRNA and antibody - AB) on body mass, head-body length, sexual maturity and fertility in *M. natalensis* infected by Lassa (LASV), Morogoro (MORV) and Gairo (GAIV) viruses. The last two columns represent animals that were vRNA or antibody positive. *Abbreviations*: *n*, number of sampled *M. natalensis*; *, statistically significant; NP^a^, not possible to calculate because of significant interaction with ELW; NP^b^, not possible to calculate because there were not enough data sampled in one of the two seasons.

|  |  |  | **season** | | **Interaction season x Lens** | | **Interaction season x vRNA** | | **Interaction season x AB** | | **Interaction season x vRNA or AB** | |
| --- | --- | --- | --- | --- | --- | --- | --- | --- | --- | --- | --- | --- |
| Virus | *Predicted* | *n* | $\chi_{1}^{2}$ | *p-value* | $\chi_{1}^{2}$ | *p-value* | $\chi_{1}^{2}$ | *p-value* | $\chi_{1}^{2}$ | *p-value* | $\chi_{1}^{2}$ | *p-value* |
| **LASV** | Body mass | 1297 | 0.97 | 0.32 | 0.05 | 0.82 | 3.88 | 0.05 | 1.34 | 0.25 | 7.23 | 0.01* |
|  | Head-body length | 1296 | 0.27 | 0.59 | 0.28 | 0.59 | 2.56 | 0.11 | 1.52 | 0.22 | 6.42 | 0.01* |
|  | Sexual maturity | 1297 | 0.32 | 0.57 | 0.51 | 0.47 | 0.32 | 0.57 | 0.99 | 0.32 | 0.89 | 0.36 |
|  | fertility | 151 | 2.17 | 0.14 | 0.96 | 0.32 | 0.21 | 0.64 | 1.14 | 0.28 | 0.41 | 0.51 |
|  | Body mass | 747 | 3.49 | 0.06 | 0.10 | 0.75 | 1.24 | 0.27 | 0.01 | 0.94 | 0.13 | 0.72 |
| **MORV** | Head-body length | 514 | NP^b^ | NP^b^ | NP^b^ | NP^b^ | NP^b^ | NP^b^ | NP^b^ | NP^b^ | NP^b^ | NP^b^ |
|  | Sexual maturity | 749 | NP^a^ | NP^a^ | 7.64 | 0.01 | 0.13 | 0.71 | 0.26 | 0.61 | 0.78 | 0.38 |
|  | Body mass | 258 | NP^b^ | NP^b^ | NP^b^ | NP^b^ | NP^b^ | NP^b^ | NP^b^ | NP^b^ | NP^b^ | NP^b^ |
| **GAIV** | Head-body length | 254 | NP^b^ | NP^b^ | NP^b^ | NP^b^ | NP^b^ | NP^b^ | NP^b^ | NP^b^ | NP^b^ | NP^b^ |
|  | Sexual maturity | 256 | NP^b^ | NP^b^ | NP^b^ | NP^b^ | NP^b^ | NP^b^ | NP^b^ | NP^b^ | NP^b^ | NP^b^ |

**Additional file 1: Table S3**

Relation between viral infection (viral RNA - vRNA and antibody - AB) and age (eye lens weight) in *M. natalensis* infected by Lassa (LASV), Morogoro (MORV) and Gairo (GAIV) viruses.

|  |  | **vRNA** | | **AB** | | **Interaction AB x vRNA** | |
| --- | --- | --- | --- | --- | --- | --- | --- |
|  | *n* | $\chi_{1}^{2}$ | *p-value* | $\chi_{1}^{2}$ | *p-value* | $\chi_{1}^{2}$ | *p-value* |
| **LASV** | 1293 | 1.09 | 0.29 | 56.48 | <0.01* | 36.20 | <0.01* |
| **MORV** | 739 | 20.15 | <0.01* | 0.55 | 0.45 | 4.76 | 0.03* |
| **Gairo** | 255 | 1.93 | 0.16 | 13.55 | <0.01* | 7.64 | <0.01* |

**Additional file 1: Table S4**

Effects on body mass, head-body length and sexual maturity of *M. natalensis* between a Lassa virus (LASV) positive and negative village (Brissa versus Tambaya) (*n* = number of sampled *M. natalensis*).

|  |  |  | **Village** | | **Interaction ELW x Village** | |
| --- | --- | --- | --- | --- | --- | --- |
| Virus | *Predicted* | *n* | $\chi_{1}^{2}$ | *p-value* | $\chi_{1}^{2}$ | *p-value* |
| **LASV** | Weight | 102 | 0.47 | 0.50 | 2.61 | 0.10 |
|  | Head-body length | 102 | 1.65 | 0.19 | 1.07 | 0.30 |
|  | Sexual maturity | 102 | 2.58 | 0.11 | 0.32 | 0.57 |
