# Supplementary material for: No measurable adverse effects of Lassa, Morogoro and Gairo arenaviruses on their rodent reservoir host in natural conditions
Source: Parasit Vectors. 2017 Apr 27;10:210. doi: 10.1186/s13071-017-2146-0 (PMC5408478; doi:10.1186/s13071-017-2146-0)
Supplement: Supplementary file 2 — Relationships between body mass, head-body length, sexual maturity (1 = active and 0 = inactive) and Eye lens weight (as a proxy for age) in M. natalensis infected by Lassa virus (LASV). Red = LASV positive village (Brissa), black = LASV negative village (Tambaya). Dashed lines represent standard errors on the predicted probabilities. (PDF 100 kb) [file 13071_2017_2146_MOESM2_ESM.pdf]

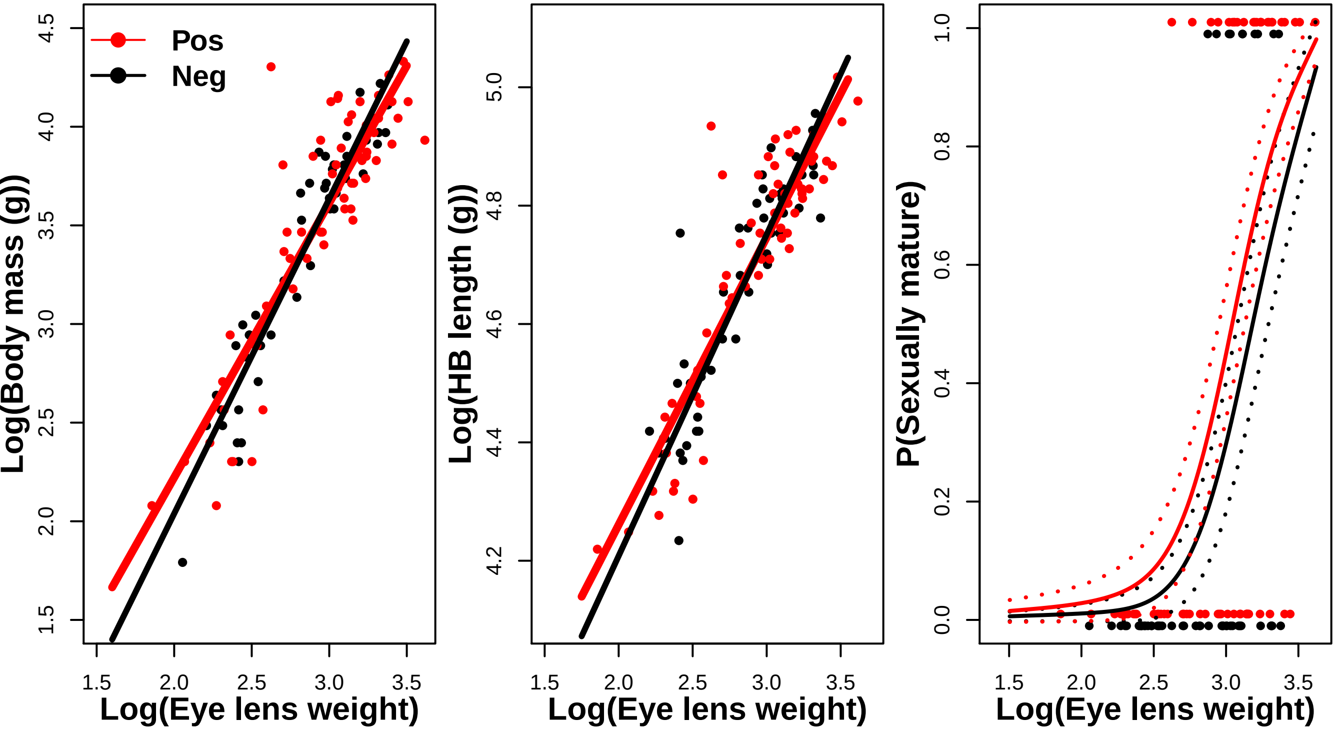

Pos
Neg
Log(Body mass (g))
Log(HB length (g))
P(Sexually mature)
Log(Eye lens weight)
Log(Eye lens weight)
Log(Eye lens weight)
1.5
2.0
2.5
3.0
3.5
4.0
4.5
4.2
4.4
4.6
4.8
5.0
0.0
0.2
0.4
0.6
0.8
1.0

**Additional file 2**

**Additional file 2: figure S1:** Relationships between body mass, head-body length, sexual maturity (1 = active and 0 = inactive) and Eye lens weight (as a proxy for age) in *M. natalensis* infected by Lassa virus (LASV). *Red* = LASV positive village (Brissa), *black* = LASV negative village (Tambaya). Dashed lines represent standard errors on the predicted probabilities.
